# Chitosan Elicitation Impacts Flavonolignan Biosynthesis in *Silybum marianum* (L.) Gaertn Cell Suspension and Enhances Antioxidant and Anti-Inflammatory Activities of Cell Extracts

**DOI:** 10.3390/molecules26040791

**Published:** 2021-02-03

**Authors:** Muzamil Shah, Hasnain Jan, Samantha Drouet, Duangjai Tungmunnithum, Jafir Hussain Shirazi, Christophe Hano, Bilal Haider Abbasi

**Affiliations:** 1 Department of Biotechnology, Quaid-i-Azam University, Islamabad-45320, Pakistan; mshah@bs.qau.edu.pk (M.S.); rhasnain849@gmail.com (H.J.); 2 Laboratoire de Biologie des Ligneux et des Grandes Cultures (LBLGC), University of Orleans, INRAE USC1328, F28000 Chartres, France; samantha.drouet@univ-orleans.fr; 3 Department of Pharmaceutical Botany, Faculty of Pharmacy, Mahidol University, 447 Sri-Ayuthaya Road, Rajathevi, Bangkok 10400, Thailand; duangjai.tun@mahidol.ac.th; 4 Department of Pharmacy, Islamia University of Bahawalpur, Bahawalpur 63100, Pakistan; jafir.shirazi@iub.edu.pk

**Keywords:** antioxidant, anti-inflammatory, chitosan, flavonoids, phenolics, *Silybum marianum*

## Abstract

*Silybum marianum* (L.) Gaertn is a rich source of antioxidants and anti-inflammatory flavonolignans with great potential for use in pharmaceutical and cosmetic products. Its biotechnological production using in vitro culture system has been proposed. Chitosan is a well-known elicitor that strongly affects both secondary metabolites and biomass production by plants. The effect of chitosan on *S. marianum* cell suspension is not known yet. In the present study, suspension cultures of *S. marianum* were exploited for their in vitro potential to produce bioactive flavonolignans in the presence of chitosan. Established cell suspension cultures were maintained on the same hormonal media supplemented with 0.5 mg/L BAP (6-benzylaminopurine) and 1.0 mg/L NAA (α-naphthalene acetic acid) under photoperiod 16/8 h (light/dark) and exposed to various treatments of chitosan (ranging from 0.5 to 50.0 mg/L). The highest biomass production was observed for cell suspension treated with 5.0 mg/L chitosan, resulting in 123.3 ± 1.7 g/L fresh weight (FW) and 17.7 ± 0.5 g/L dry weight (DW) productions. All chitosan treatments resulted in an overall increase in the accumulation of total flavonoids (5.0 ± 0.1 mg/g DW for 5.0 mg/L chitosan), total phenolic compounds (11.0 ± 0.2 mg/g DW for 0.5 mg/L chitosan) and silymarin (9.9 ± 0.5 mg/g DW for 0.5 mg/L chitosan). In particular, higher accumulation levels of silybin B (6.3 ± 0.2 mg/g DW), silybin A (1.2 ± 0.1 mg/g DW) and silydianin (1.0 ± 0.0 mg/g DW) were recorded for 0.5 mg/L chitosan. The corresponding extracts displayed enhanced antioxidant and anti-inflammatory capacities: in particular, high ABTS antioxidant activity (741.5 ± 4.4 μM Trolox C equivalent antioxidant capacity) was recorded in extracts obtained in presence of 0.5 mg/L of chitosan, whereas highest inhibitions of cyclooxygenase 2 (COX-2, 30.5 ± 1.3 %), secretory phospholipase A2 (sPLA2, 33.9 ± 1.3 %) and 15-lipoxygenase (15-LOX-2, 31.6 ± 1.2 %) enzymes involved in inflammation process were measured in extracts obtained in the presence of 5.0 mg/L of chitosan. Taken together, these results highlight the high potential of the chitosan elicitation in the *S. marianum* cell suspension for enhanced production of antioxidant and anti-inflammatory silymarin-rich extracts.

## 1. Introduction

*Silybum marianum* belongs to the family Asteraceae, generally known as its common name, milk thistle, and is an essential medicinal herb with strong hepatoprotective activity [1]. *S. marianum* demand per year varies from 18 to 20 t, while its annual sale is about 8 billion USD [2]. The prominent component of *S. marianum* is silymarin an isomeric mixture of various flavonolignan analogues like silybins, isosilybins, silychristin and silydianin together with the flavonoid taxifolin [3,4]. Silymarin neutralizes the effect of oxidative damage due to high free radical scavenging activity, thereby protecting human hepatic tissue [5]. Both in vitro and in vivo experiments on living models have shown that silymarin plays a protective role against toxins in hepatic cells [4,6]. Silymarin exhibits numerous medicinal properties, including anti-arthritic, anti-cancer, anti-diabetic, anti-viral and immunomodulatory [4,7,8,9,10], and is beneficial in the treatment of obsessive compulsive disorder (OCD), *β*-thalassemia and non-alcoholic liver fat disorder (NAFLD) [11,12,13]. Among the most desirable biological activities, its antioxidant and anti-inflammatory activities are well described [4,7,14,15].

In human cells, although mechanisms exist for repairing oxidatively damaged biomolecules, some damage remains. The theory of free radical aging assumes from this observation that reactive oxygen and nitrogen species (ROS/RNS) can induce oxidative damage, cause cell dysfunction and physiological deterioration, leading to aging, the emergence of degenerative diseases, and ultimately death [16]. Plants produce numerous active compounds during growth, such as phenolics that serve as natural protective antioxidant agents [16,17]. The redox properties of molecules are usually considered responsible for the antioxidant activities [16,18], which allow them to act as reducing agents or donor of hydrogen atoms [19]. Inflammation is another major player in the emergence of degenerative diseases. Plant extracts are a common natural sources of anti-inflammatory compounds [20,21]. In general, their anti-inflammatory capacity is determined by their ability to inhibit key enzymes involved in the inflammation process such as COX-1 (cyclooxygenase 1), COX-2 (cyclooxygenase 2), sPLA2 (secretory phospholipase A2) and 15-LOX-2 (15-lipoxygenase). In particular, COXs are the key players and the targets for the development of non-steroidal anti-inflammatory drugs in the inflammation process. The COX-1 and COX-2 enzymes are generally both inhibited by anti-inflammatory drugs. Prostaglandin E2 is produced by COX-2, the endogenous pain causing molecule. COXs also help to maintain platelet and kidney homeostasis, gastrointestinal tissue homeostasis, and are expressed in different forms of cancer [22]. Drugs that block both COX-1 and COX-2 enzymes can therefore cause detrimental side effects, such as renal impairment and/or gastrointestinal bleeding. Thus, researchers are now looking for the right candidates for drug development that can only inhibit COX-2 [16]. *S. marianum* flavonolignans, silychristin in particular, showed anti-inflammatory activity though their COX-1 inhibition capacity [14,15]. The anti-inflammatory action of silymarin have been also demonstrated in patients with type 2 diabetes mellitus [23]. It has been reported that silymarin, either used alone or in combination with non-steroidal anti-inflammatory drugs, is able to decrease interleukins levels and complemented proteins in patients with knee osteoarthritis [24]. Recently, the in vitro anti-inflammatory through the inhibitions of COXs, sPLA2 and 15-LOX-2 of *S. marianum* extracts from in vitro callus cultures was also reported [3].

*S. marianum* accumulates very attractive antioxidant and anti-inflammatory components with considerable potential for use in both pharmaceutical and cosmetic products [3]. Traditional cultivation of milk thistle plants is vulnerable to many problems, especially because of the spiny nature of the flowers and leaves, leading to a reduction in total yield. In addition, the use of herbicides results in the fruits getting contaminated with toxins. Due to the increase interests in silymarin, alternative and sustainable bioproduction platforms other than conventional cultivation of plants for their production are needed. Biotechnological in vitro propagation could be extremely useful for solving these complications. Moreover, plant tissue and cell culture techniques may be used to boost the biosynthesis of such metabolites. Several abiotic and biotic elicitors have previously been used in vitro to increase secondary metabolite content in medicinal plant species. Elicitors modify plant metabolism by causing physiological stress that contribute to the stimulation of phytochemical biosynthesis [25,26]. Chitosan is a biotic elicitor affecting in vitro numerous physiological processes like photosynthesis, hence morphogenesis, development and growth of various medicinal plants [27,28]. Chitosan is a polycationic *β*-1,4 linked d-glucosamine polymer that acts as an antifungal agent [29] through the phytoalexin production stimulation [30] and pathogenesis-related proteins elicitation in the host [31]. Chitosan has been reported to stimulate silymarin accumulation in milk thistle callus cultures [32].

In the present study, chitosan, applied at different concentration levels, is evaluated for the stimulation of silymarin biosynthesis in *S. marianum* cell suspension, resulting in extracts with enhanced anti-inflammatory and antioxidant potential. The present study reports on silymarin enhancement in the suspension culture of *S. marianum* using chitosan. The quantification of silymarin was obtained by a validated high-performance liquid chromatography method. Using both in vitro assays with different mechanisms and cellular assays, antioxidant activity of each was evaluated. The anti-inflammatory potential of each extract has been determined to inhibit the COX-1, COX-2, 15-LOX and sPLA2 enzymes.

## 2. Results and Discussion

### 2.1. Effect of Chitosan on Biomass Accumulation

*S. marianum* cell suspension culture was established from leaf-derived callus previously obtained in Murashige and Skoog (MS) [33] medium supplemented with 0.5 mg/L BAP (6-benzyl aminopurine) and 1 mg/L NAA (α-naphthalene acetic acid), and was subjected to different concentration levels of chitosan. The effect of the different chitosan treatments on biomass production was first assessed on the basis of both fresh weight (FW) and dry weight (DW) measurements. A significant variation in accumulation of biomass was observed by employing different chitosan concentrations (Figure S1). The biomass production appeared to be dependent on the concentration of chitosan used, with a stimulatory effect up to 5.0 mg/L chitosan, and then a decrease in biomass production at higher concentrations used (Table 1). Maximum accumulation of biomass (both FW of and DW) was obtained under a chitosan concentration of 5 mg/L (MCH5) compared to the control condition (MCH1) (Table 1).

In our previous works, we show that callus cultures can be a promising system for the production of antioxidant and anti-inflammatory *S. marianum* extracts [3]. Callus is one of the most important steps in initiating an in vitro culture, but for industrial applications, their growth rate, genetic uniformity and/or stability sometimes leading to erratic production of secondary metabolites, and difficulty in scale-up are often difficult to resolve [34]. Cell suspension cultures are more commonly used for industrial purposes due to their uniformity, resulting in more stable production, rapid growth and ease of scale-up for biomass production [34]. Elicitation is an effective strategy to activate the production of bioactive metabolites [34]. However, most of the biotic elicitors are commonly found to have a detrimental effect on plant biomass accumulation [34]. Depending on the concentration, chitosan may have a negative impact on plant biomass accumulation, but this negative impact is counterbalanced by its beneficial nutritional action [35,36,37,38,39,40,41,42]. Increased biomass accumulation following chitosan application results from its ability to boost the availability and absorption of water and essential nutrients by controlling the cell osmotic pressure [43,44]. Stimulation of biomass production has been reported for various in vitro culture systems for different plant species such as cell suspensions of different basil species [41] and red sage (*Salvia miltiorhiza*) [45], callus cultures of flax [39] and *Fagonia indica* [40], as well as adventitious roots of Indian ginseng (*Withania somnifera*) [31] when treated with chitosan. On the opposite, the decreased biomass accumulation for the elevated concentrations of chitosan applied is the result of its elicitor action.

### 2.2. Total Phenolic and Flavonoid Contents in S. marianum Cell Suspension Extract (SMCE)

**Figure 1:**
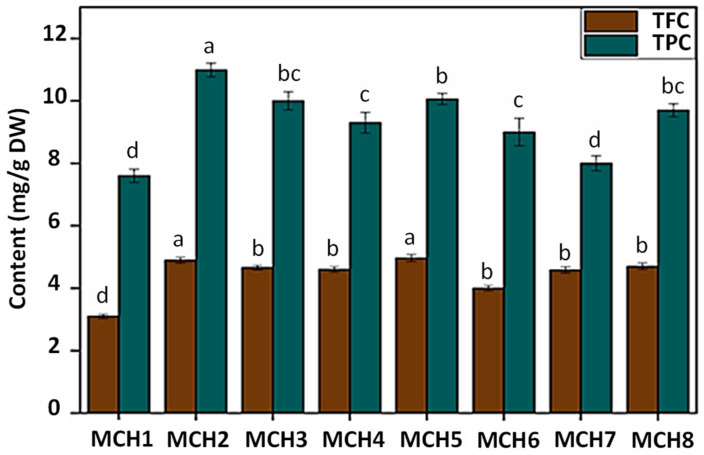
Phenolic and flavonoid content of samples at different chitosan concentrations. MCH1: control; MCH2: 0.5 mg/L chitosan; MCH3: 1.0 mg/L chitosan; MCH4: 2.5 mg/L chitosan; MCH5: 5.0 mg/L chitosan; MCH6: 10.0 mg/L chitosan; MCH7: 25.0 mg/L chitosan; MCH8: 50.0 mg/L chitosan; Values are means ± SD of three independent replicates. Different letters represent significant differences between the various extraction conditions (*p* < 0.05).

Total flavonoid (TFC) and total phenolic (TPC) content were determined to estimate the effects of chitosan elicitation on the production of secondary metabolites in *S. marianum* cell suspension (Figure 1). An increase in both total flavonoid and phenolic contents was observed for all chitosan treatments. *S. marianum* cell suspension extract (SMCE) obtained under condition MCH5 (5.0 ± 0.1 mg/g DW) was found to have the highest TFC followed by condition MCH2 (4.9 ± 0.1 mg/g DW) (Figure 1). Similarly, highest TPC (11.0 ± 0.2 mg/g DW) was observed in SMCE corresponding to condition MCH2 followed by condition MCH5 (10.1 ± 0.2 mg/g DW) (Figure 1).

In the present study, chitosan elicitation leads to the stimulation of the accumulation of these metabolites. Plants have evolved a defense system based on a wide range of molecules that lead to growth and survival in response to various environmental factors, including abiotic and biotic pressures. Phytochemicals, such as flavonoids and phenolic compounds, can be formed under unfavorable circumstances [46,47]. A classic biotechnological approach to increase the production of bioactive secondary metabolites is based on the use of elicitors that activate secondary metabolic pathways to promote plant defense [34]. As a biotic elicitor with limited negative impact on plant biomass production, chitosan has been widely used [35,36,37,38,39,40,41,42]. In accordance with our observations, its stimulating impact on the production of phenylpropanoids has already been reported in several plant species [35,36,37,38,39,40]. This can result, in particular, from the mentioned activation of key enzymes of the phenylpropanoid pathway such as l-phenylalanine ammonia lyase (PAL) or chalcone synthase (CHS) [35,36,37,38,39,40,48].

### 2.3. Chitosan Effect on Silymarin Accumulation

Using HPLC analysis, a more complete view of the phytochemistry was obtained by quantifying the individual composition of silymarin in extracts collected after treatment with different chitosan concentrations (Table 2).

The highest total silymarin content was obtained under conditions MCH2 and MCH5 compared to control (i.e., MCH1). High performance liquid chromatography (HPLC) analysis of silymarin individual compounds has shown that silybin B, silydianin and silybin A are the main phytochemicals produced by suspension cultures of *S. marianum*. Chitosan showed a stimulatory effect, in particular on silybin B and silybin A. On the opposite, the taxifolin accumulation was very low for all the conditions. Since the main flavonolignans from *S. marianum* are synthesized from taxifolin [49], it could be assumed that this low level of taxifolin might be due to the result of its conversion into other flavonolignans [50,51]. Several biotic elicitors deriving from fungal cell wall such as chitosan and yeast extract have been reported to stimulate silymarin production [48,52,53]. This activation was related to the ability of these elicitors to induce the CHS enzyme activity [48], which was shown to be closely related to the accumulation of silymarin in *S. marianum* [54]. Chitosan has also been reported to induce PAL at both gene expression and/or enzymatic levels in several plant species [35,36,37,38,39,40]. The PAL enzyme is the point of entry of l-phenylalanine into the phenylpropanoid pathway. In plant defense mechanisms, this enzyme is considered to play a key role, and is generally responsible for the increased carbon flux through this pathway, contributing to an increased biosynthesis of defense/stress-related compounds derived from the phenylpropanoid pathway [55]. In particular, fungal elicitors have been shown to stimulate the production of monolignols and monolignol-derived products [56,57]. As hybrid compounds composed of flavonoid and monolignol moities, flavonolignans may benefit from the stimulation of PAL and CHS contributing to the formation of these precursors [54]. The differential induction of the different flavonolignans may result from competition for these precursors as well as from a distinct biosynthetic route or regulation. Indeed, it has been suggested that the biosynthesis of these flavonolignans can require different and possibly complex oxidative coupling mechanisms that could imply the intervention of peroxidase(s) or even laccase(s) [54,58], but also dirigent proteins, as already described in many plant species, in order to guide the stereoselective and/or regioselective biosynthesis of many lignan derivatives [59,60,61].

### 2.4. Effect of Chitosan on Antioxidant Activities of SMCE

In the present study, the antioxidant capacity of the SMCEs derived from *S. marianum* suspension culture in response to various chitosan treatments has been explored by the use of four different antioxidant assays. Three in vitro assays based on distinct mechanisms (DPPH, ABTS and FRAP) were used. DPPH assay is based on both ET- (single electron transfer) and HAT- (hydrogen atom transfer) antioxidant mechanism, and was expressed as percentage of free radical scavenging activity (% FRSA). ABTS (HAT-based antioxidant mechanism) and FRAP (ET-based antioxidant mechanism) were expressed as trolox C equivalent antioxidant capacity (µM TEAC). The cellular assay relied on the evaluation of ROS and RNS production in yeast cells subjected to UV-induced oxidative stress, and was expressed as inhibition percentage of RO/NS production (Table 3).

The highest DPPH antioxidant activity (90.4 ± 1.0 % FRSA) was recorded for SMCE derived from condition MCH5 followed by condition MCH8 (89.5 ± 0.9 %FRSA). Only slight differences were observed between the FRAP antioxidant activity of the various samples with the highest recorded extract activity resulting from the MCH2 condition (334.5 ± 3.3 µMTEAC). The highest ABTS antioxidant activity was shown for SMCE derived from condition MCH2 (741.5 ± 4.4 µM TEAC) followed by condition MCH5 (730.4 ± 4.6 µM TEAC) (Table 3). This increased in vitro antioxidant activity was confirmed in cellulo in yeast model under UV-induced oxidative stress. In this cellular antioxidant assay, the highest inhibition of ROS production was observed for SMCE derived from conditions MCH52 (78.8 ± 1.3 % of ROS inhibition) and MCH6 (78.4 ± 1.2 % of ROS inhibition) (Table 3).

The sudden shift in plant metabolic pathways due to environmental stress results in the production of reactive oxygen species that can damage plant cells, proteins, membrane lipids and DNA [62,63,64]. A number of metabolic compounds that function as a protective mechanism, such as terpenoids, phenolic and flavonoids, are produced by plants in response to oxidative stress and a strong association is generally observed between these secondary metabolites and antioxidant activity [65,66,67]. Here, in suspension cultures of S. marianum, chitosan improved the accumulation of phenolic compounds, which consequently improved its ability for antioxidants. In general, the antioxidant potential of phenolic profiling has been widely described in many plant species [68,69]. Several studies have documented the potential role of silymarin in reducing the production of reactive oxygen species through the scavenging of free radicals [70,71,72].

### 2.5. The Effect of Chitosan on the Anti-Inflammatory Activity

Various in vitro assays such as COX-1, COX-2, 15-LOX and sPLA were conducted to explore the potential of the present SCME as potent anti-inflammatory agents. The resulting percentage inhibition for each assay are shown in Table 4. The maximum inhibitory activities were recorded against 15-LOX (35.4 ± 1.3 %) followed by sPLA2 (34.2 ± 0.9 %) for SMCE obtained from suspension cultures grown under condition MCH2 (Table 3). Interestingly, a more substantial inhibition of COX-2 rather than COX-1 for all SCME was observed with maximum inhibition recorded for the extract resulting from condition MCH2 (31.2 ± 1.0 %).

The anti-inflammatory action is exerted with often differential action on COX-1, COX-2, 15LOX and sPLA2, thus reducing concentrations of prostanoid and leukotrienes [73]. The in vitro anti-inflammatory activities of many phenylpropanoids have been identified via multiple pathways such as COX inhibition [20,74,75]. It has been evidenced previously that anti-inflammatory activity of S. marianum relied on silymarin content [76,77,78]. Similarly, Pradhan et al. [79] also found that increased silymarin production enhances anti-inflammatory activity.

### 2.6. Correlation Analysis

**Figure 2:**
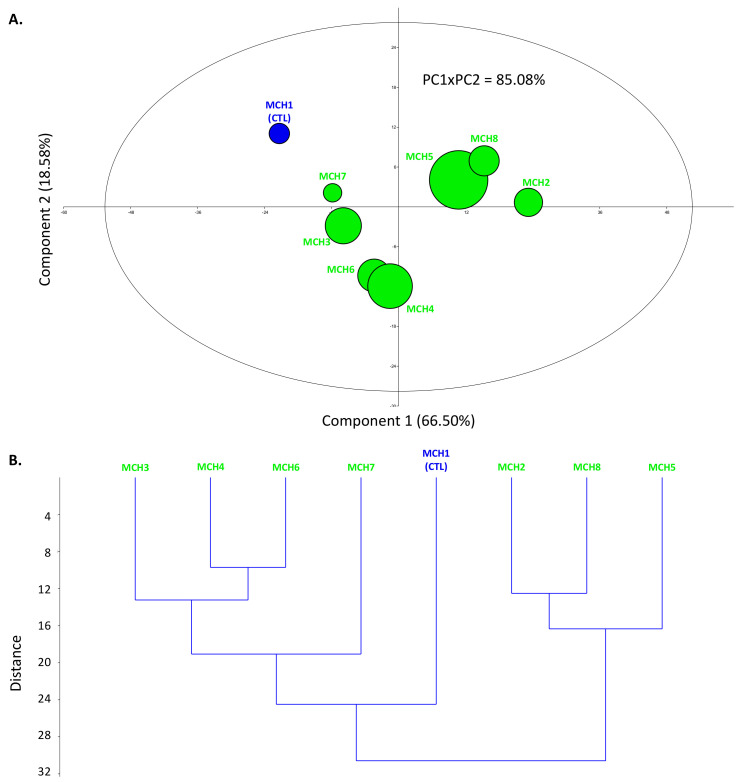
(**A**) Principal component analysis (PCA) for the discrimination of the different SMCE as a function of their phytochemical compositions and biological activities with round size relative to the biomass expressed as dry weight. Variance of factor 1 (PC1) = 66.50% and of factor 2 (PC2) = 18.58%. (**B**) Hierarchical clustering analysis (HCA) for the descrimination of the different SMCE as a function of their phytochemical compositions and biological activities (method used: paired groups with similarity measured using Euclidian distance between each group). MCH1: control; MCH2: 0.5 mg/L chitosan; MCH3: 1.0 mg/L chitosan; MCH4: 2.5 mg/L chitosan; MCH5: 5.0 mg/L chitosan; MCH6: 10.0 mg/L chitosan; MCH7: 25.0 mg/L chitosan; MCH8: 50.0 mg/L chitosan.

Both principal component analysis (PCA) and hierarchical clustring analysis (HCA) were performed to visualize the effect of different chitosan treatments on phytochemistry and biological activity of SMCE (Figure 2).

The PCA separation explained 85.08% of the apparent complexity of the current bioproduction system (PC1 × PC2, Figure 2A). Discrimination occurred mainly through the first dimension (PC1 axis), which itself explained 66.50% of the apparent complexity and allowed the separation of the different extracts according to their phytochemical composition (in particular silybin B and total silymarin content) and biological activity (in particular ABTS-based antioxidant activity as well as anti-inflammatory activities) (Figure S2A). The second axis (PC2) accounted for 18.58% of the initial variability, but allowed for a clear discrimination between the effect of the different chitosan treatments on biological activities, with a major influence on the antioxidant activity mechanism (Figure 2A, Figure S2B).

HCA confirmed the impact of chitosan treatments (Figure 2B). Indeed, a significant distance between the control and the chitosan-treated cell suspension was observed (Figure 2B), as already shown by the PCA (Figure 2A). The effect of chitosan on the production of biomass, as shown in Figure 2A, appeared to be complex depending on the concentration of chitosan added to the cell suspension. This may be related to the chitosan structure as a polycation polymer of β-1,4-glucosamine, which may act either as a biotic elicitor or as a fertilizer that supplies sugar and nitrogen to plants [29,39].

**Figure 3:**
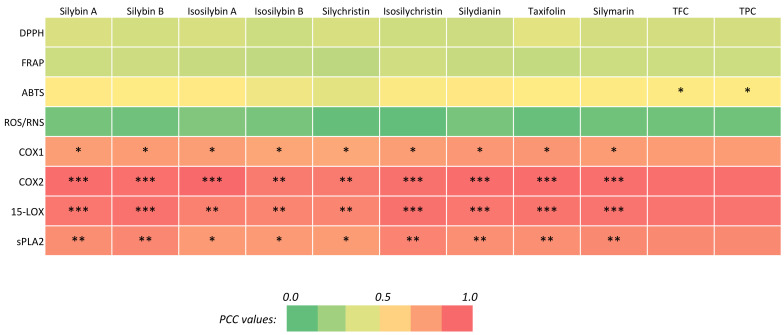
Correlation analysis (PCC) of the relation between the main phytochemicals from *S. marianum* cell suspension extracts and the antioxidant (in vitro DPPH, ABTS and FRAP assays, and cellular RO/NS production) and anti-inflammatory (COX-1, COX-2, 15-LOX and sPLA2) activities. Significance level: * *p* < 0.05, ** *p* < 0.01, *** *p* < 0.001. PCC values are indicated in Table S1.

Correlation analysis (using Pearson coefficient correlation, PCC) showed a higher correlation of different phytochemicals with anti-inflammatory activity than with antioxidant capacity (Figure 3, Table S1).

A significant correlation between the results of the ABTS assay and the total accumulations of flavonoids and phenolic compounds was noted for antioxidant activity (PCC = 0.782 and *p* = 0.022 for TFC, and PCC = 0.815, *p* = 0.014 for TPC) (Figure 3; Table S1). A similar trend indicating a higher linear association of HAT-based antioxidant assays with phenolic compounds than with flavonoids has already been reported [80]. On the contrary, the different phytochemicals did not individually showed any significant correlation with antioxidant assays (Figure 3, Table S1). As already observed with some extracts, this may result from synergistic activity between different compounds (cocktail effect), which may be more efficient than a single compound in preventing oxidative stress [81]. In addition, it is not excluded that other types of phenolics (not flavonolignans) may have more antioxidant capacity than silymarin.

In contrast, individual compounds displayed a high and significant correlation with all assays for anti-inflammatory activity (Figure 3, Table S1). The different PCCs ranged: (i) for COX-1 inhibition, from 0.770 (*p* = 0.025) to 0.823 (*p* = 0.011) for taxifolin and silychristin, respectively; (ii) for COX-2 inhibition, from 0.970 (*p* < 0.0001) to 0.913 (*p* = 0.0016) for isosilybin B and isosilybin A, respectively; (iii) for 15-LOX inhibition, from 0.878 (*p* = 0.004) to 0.946 (*p* = 0.0004) for silychristin and isosilychristin, respectively; and (iv) for sPLA2 inhibition, from 0.808 (*p* = 0.0015) to 0.886 (*p* = 0.0034) for silychristin and isosilychristin, respectively (Figure 3, Table S1). Our results align well with recent work showing the anti-inflammatory activity of *S. marianum* flavonolignans and, in particular, the strongest silychristin COX-1 inhibition capacity compared to other flavonolignans [14,15]. These results confirmed the anti-inflammatory capacity of *S. marianum* extracts from in vitro cultures [3]. Interestingly, a higher inhibition potential for COX-2 than COX-1 is observed here with our SMCE compared to our previous study using callus cultures [3], which is of particular interest in the current search of selective inhibitors [16]. The antioxidant and anti-inflammatory capacities of silymarin have been reported previously in patients with type 2 diabetes mellitus [23]. Similarly, reports have shown that silymarin either used alone or in combination with non-steroidal anti-inflammatory drugs decreases the high levels of interleukins or complemented proteins in patients with knee osteoarthritis [24].

## 3. Material and Methods

### 3.1. Seed Collection and Germination Conditions

Seeds of *S. marianum* were collected from the Mardan (natural habitat) division of Khyber Pu-khtoonkhawa (Pakistan), certified by a botanist and deposited at the Plant Cell Culture Lab seed bank (Department of Biotechnology, Quaid-i-Azam University, Pakistan). For germination, the seed surfaces were sterilized using 70% ethanol and 0.1% mercuric chloride for 90 s and 40 s, respectively, followed by 3 times washing with sterile distilled water, and sterilized filter paper was used for drying. Previously established protocol [3] was used for inoculation of the sterilized seeds on Murashige and Skoog (MS) [33] basal medium. Growth room having 16/8 h (light/dark) photoperiod with 40µmol/m^2^ /s light intensity (dark red/white LED (18 W, Green Power TLED DR/W, Philips), relative humidity (RH) of 30% and maintained temperature at 25 ± 2 °C.

### 3.2. Callus Culture Initiation

The 4-week-old leaves from in vitro plantlets were excised for callus initiation. The streamlined protocol [3] has been used for explant inoculation. Leaf explants (0.5 cm^2^) were incubated on solid MS-derived media (supplemented with BAP 0.5 mg/L, NAA 1.0 mg/L, sucrose (30 g/L), agar (8 g/L) and pH 5.6–5.8). The leaf-derived calli obtained after 4 weeks incubation in growth room (16/8 h (light/dark) photoperiod with 40µmol/m^2^ /s light intensity (dark red/white LED (18 W, Green Power TLED DR/W, Philips), relative humidity (RH) of 30% and temperature of 25 ± 2 °C). Callus cultures were then sub-cultured each 2 weeks to ensure 100% homogeneity of the callus culture.

### 3.3. Cell Suspension Culture Initiation

Homogeneous leaf-derived calli were inoculated for cell suspension culture in Erlenmeyer flasks (250 mL) containing liquid MS media with BAP 0.5 mg/L, NAA 1.0 mg/L, and sucrose 30 g/L. Flasks were kept in 16/8 h (light/dark) photoperiod with 40 µm/m^2^ /s light intensity at temperature 25 ± 2°C on gyratory shaker at constant agitation (120 rpm) for 2 weeks. For preparation of inoculum, Erlenmeyer flask containing 100 mL of the MS-derived medium and 1 g FW callus were used.

### 3.4. Elicitor Preparation and Treatments

Chitosan (C_611_NO_4_) (Merck Chemicals, Saint-Quentin Fallavier, France) was used for elicitation (deacetylating grade: 70–85 %). Chitosan was dissolved in 0.1% acetic acid at 50 °C with constant stirring for 5 h. Different concentration levels (0.5, 1.0, 2.5, 5.0, 10, 20, 50 mg/L) of chitosan have been introduced to the MS-derived culture medium. The same volume (1 mL of each chitosan solution dissolved in MS medium) was added to each MS-derived medium. Medium without chitosan addition (addition of 1 mL of fresh MS medium) was used as control (Table 5).

To execute the experiment, Erlenmeyer flasks containing 40 mL of media and 400 mg FW callus were used. Cell suspension cultures were maintained in 16/8h (light/dark) photoperiods at 120 rpm on gyratory shaker and a temperature of 25 ± 2 °C for 14 days. Each experiment was performed in triplicate.

### 3.5. Biomass Determination

Cell cultures were harvested for determination of fresh weight (FW) and filtered using 0.45 µm stainless steel sieves (Merck Chemicals, Saint-Quentin Fallavier, France). Cell cultures were then gently washed with double distilled water, dried using sterile filter paper sheets for removal of water excess, and then weighed for FW determination using a precision balance (Mettler Toledo, Viroflay, France). For dry weight (DW) estimation, cells were frozen and lyophylized 48 h (lyophilizator CHRIST Alpha 1–5, Martin Christ Gefriertrocknungsanlagen GmbH, Osterode am Harz, Germany) and then weighed using a precision balance (Mettler Toledo, Viroflay, France).

### 3.6. Preparation of the S. marianum Cell Suspension Extracts (SCMEs)

Extracts were prepared using the validated method developed for silymarin extraction [49]. One hundred mg DW of each cell suspension was extracted in 2.5 mL of 54.5 % (*v*/*v*) aqueous EtOH using ultrasound at a frequency of 36.6 kHz during 60 min at 45 °C. The characteristic of the ultrasonic bath (USC1200TH, Prolabo, Fontenay-sous-Bois, France) are: inner dimensions of 300 mm × 240 mm × 200 mm, maximal heating power 400W (acoustic power of 1W/cm^2^), equipped with a digital timer, a frequency and a temperature controller. Prior to HPLC analysis, each extract was centrifuged at 10,000 rpm (12,520× *g*) for 10 min (Heraeus Megafuge 16R, Hanau, Germany) and the supernatant was filtered through 0.45 µm nylon syringe membranes (Macherey Nagel, Hoerdt, France). Extracts were stored at −20 °C before phytochemical and biological evaluations.

### 3.7. Determination of Total Phenolic Content (TPC)

According to the previous protocol, total phenolic content (TPC) was calculated using the Folin-Ciocalteu (FC) reagent. FC reagent (90 μL) and sodium carbonate (90 μL) were combined with sample extract (20 μL). Absorbance at 725 nm was determined using a microplate reader (Synergy II, BioTek Instruments, Colmar, France) after incubation for 5 min at 25 ± 2 °C. The calibration standard used was gallic acid and TPC were expressed as gallic acid equivalents (GAE)/g DW [82].

### 3.8. Determination of Total Flavonoid Content (TFC)

The aluminum chloride colorimetric method [83,84] with minor changes was used to measure TFC. The reaction mixture consists of aluminum chloride (10 µL), sample (20 µL), potassium acetate (10 µL) and water (160 µL) to make final volume of 200 µL. The mixture was incubated for 30 min at 25 ± 2 °C and then absorbance at 415 nm was measured using a microplate reader (Synergy II, BioTek Instruments, Colmar, France). The calibration standard used was quercetin and TFC were expressed as quercetin equivalents (QE)/g of DW [85].

### 3.9. HPLC Analysis

Flavonolignans and taxifolin were quantified by HPLC analysis conducted with a Varian HPLC PAD system (Prostar 230 pump, Metachem Degasit, Prostar 410 autosampler, Prostar 335 Photodiode Array Detector (PAD) driven by Galaxie version 1.9.3.2 software (Varian, Les Ulis, France)). Separation was performed using the validated method designed for silymarin separation at 35 °C with a core-shell column (Kinetex 5 µm XB-C18, 100 Å, LC Column 150 x 4.6 mm, C18 with iso-butyl side chains, and with TMS endcapping, core-shell silica, Phenomenex Le Pecq France) [86]. A linear gradient: from an A:B 10:90 (*v/v*) to 100:0 (*v/v*) composed of methanol (A) and 0.05% formic acid acidified water (B) was applied at a flow rate of 1.00 mL/min. The injection volume was 10 µL. Quantification was done at 280 nm using calibration curves of authentic commercial standards (Merck Chemical, Saint-Quentin Fallavier, France) (linear range: 0.5–50 µg/mL; taxifolin (y = 1292.9x + 0.7; R^2^ = 0.9989; LOD = 0.09 µg/mL; LOQ = 0.26 µg/mL); silychristin (y = 2266.4x – 12.7; R^2^ = 0.9994; LOD = 0.05 µg/mL; LOQ = 0.17 µg/mL); silydianin (y = 1,649.8x + 34.8; R^2^ = 0.9992; LOD = 0.10 µg/mL; LOQ = 0.32 µg/mL); silybin A (y = 2,575.0x + 5.7; R^2^ = 0.9997; LOD = 0.05 µg/mL; LOQ = 0.15 µg/mL); silybin B (y = 2515.9x + 27.3; R^2^ = 0.9999; LOD = 0.05 µg/mL; LOQ = 0.15 µg/mL); isosilybin A (y = 2726.3x + 17.8; R^2^ = 0.9998; LOD = 0.05 µg/mL; LOQ = 0.16 µg/mL); isosilybin B (y = 2861.1x + 1.9; R^2^ = 0.9999; LOD = 0.05 µg/mL; LOQ = 0.16 µg/mL); isosilychristin was quantified using silychristin standard curve).

### 3.10. Antioxidant Activity

#### 3.10.1. DPPH Assay

Free radical scavenging assay (FRSA) using DPPH (2,2-diphenyl-1-picrylhydrazyl) was determined following the reported protocol [87] with slight modifications. Sample extract (20 µL) was mixed with 180 µL DPPH solution (3.2 mg/100 mL methanol) and the mixture was then incubated for 60 min at 25 ± 2 °C followed by dH_2_O (160 µL) addition. Absorbance microplate reader (Synergy II, BioTek Instruments, Colmar, France) was used to record the absorbance at 517 nm. To plot the calibration curve (R^2^ = 0.989) the standard used was methanolic extract 0.5 mL of DPPH solution. The free radical scavenging activity was calculated as % of discoloration of DPPH.

#### 3.10.2. FRAP Assay

FRAP (ferric reducing antioxidant power) was evaluated using protocol [87] with minor modifications. Briefly, 190 µL of FRAP (20 mM FeCl_3_ 6H_2_O, 10 mM TPTZ and 300 mM acetate buffer pH 3.6; ratio 1:1:10 (*v*/*v*/*v*)) was mixed with 10 µL of extract. After 15 min incubation at room temperature, absorbance at 630 nm was measured using a microplate reader (Synergy II, BioTek Instruments, Colmar, France). The antioxidant activity was expressed as Trolox C equivalent antioxidant capacity (TAEC).

#### 3.10.3. Antioxidant ABTS Assay

The ABTS (2,2-azinobis-3-ethylbenzthiazoline-6-sulphonic acid) assay was followed by the previously mentioned procedure [87]. In short, the solution of ABTS was prepared by combining 2.45 mM of potassium per sulphate, equal to 7 mM of ABTS salt, and the mixture was then placed in the dark for 16 h. The absorbance of the solution was measured at 734 nm (BioTek ELX800, BioTek Instruments, Colmar, France) and adjusted to 0.7 prior its used. Then, 190 µL of this ABTS solution was mixed with each extract (10 µL). The mixture was placed in the dark at room temperature (25 ± 1 °C) for 15 min and the absorbance was measured at 734 nm (Synergy II, BioTek Instruments, Colmar, France). The antioxidant activity was expressed as Trolox C equivalent antioxidant capacity (TAEC).

#### 3.10.4. Cellular Antioxidant Assay

UV-induced oxidative stress in yeast strain DBY746 (*MATα leu*2–3,112 *his*3Δ1 *trp*1-289a *ura*3-52 GAI+) grown on YPD medium was induced as described previously [80]. The level of reactive oxygen and nitrogen species (RO/NS) was determined by using the Dihydrorhodamine-123 fluorescent dye (DHR-123) [81]. Approximately 10^8^ yeast cells grown in the presence of SMCE or DMSO (control) were washed with PBS (2 times), and then resuspended in PBS solution containing 0.4 μM DHR-123 and incubated at 30 °C during 10 min in the dark. After washing with PBS (2 times), the fluorescence signal (λex = 505 nm, λem = 535 nm) was measured (VersaFluor Fluorimeter, Biorad, Marnes-la-Coquette, France).

### 3.11. Anti-Inflammatory Activities

#### 3.11.1. COX-2 and COX-1 Inhibitions

The COX-2 and COX-1 inhibitions were evaluated using the COX-2 (human) and COX-1 (Ovine) assay kit (701050, Cayman Chem. Co, Interchim, Montluçon, France) according to manufacturer recommendations, and as described previously [87]. Arachidonic acid was used as substrate, at a concentration of 1.1 mM and ibuprofen was used as a positive control at a concentration of 10 mM. Oxidized *N,N,N’,N’*-tetramethyl-*p*-phenylenediam was determined at 590 nm using microplate reader (Synergy II, BioTek Instruments, Colmar, France).

#### 3.11.2. 15-LOX Inhibition

The 15-LOX inhibition was evaluated using the assay kit (760700, Cayman Chem. Co, Interchim, Montluçon, France). The inhibitory activity of each SMCE against 15-LOX was calculated following the instructions of the manufacturer and as described previously [87]. Arachidonic acid (10 μM) was used as substrate. Nordihydroguaiaretic acid (NDGA) 100 μM was used as a positive control inhibitor. Absorbance variation at 490 nm was recorded using microplate reader (Synergy II, BioTek Instruments, Colmar, France).

#### 3.11.3. sPLA2 Inhibition

The sPLA2 inhibition was evaluated using the assay kit (10004883, Cayman Chem. Co, Interchim, Montluçon, France). The inhibitory activity of each SMCE against sPLA2 was calculated following the instructions of the manufacturer and as described previously [87]. Diheptanoyl thio-PC (1.44 μM) was used as substrate. Thiotheramide-PC (100 μM) was used as a positive control inhibitor. The free thiols released from the substrate was measured at 420 nm using microplate reader (Synergy II, BioTek Instruments, Colmar, France).

### 3.12. Statistical Analysis

Each experiment was performed in triplicates. Significant differences between groups were determined by ANOVA, followed by two-tailed multiple *t*-tests with Bonferroni correction performed with XL-STAT 2019 biostatistics software (Addinsoft, Paris, France). All results were considered significant at *p* < 0.05 represented by different letters. Principal component analysis, hierarchical clustering analysis and Pearson correlation coefficient analysis were obtained with PAST 3.0 (Øyvind Hammer, Natural History Museum, University of Oslo, Oslo, Norway) with significant thresholds at *p* < 0.05, *p* < 0.01 and *p* < 0.001 represented by *, ** and ***, respectively.

## 4. Conclusions

Cell suspension cultures of *S. marianum* were initiated and exposed to seven different concentration levels of chitosan (0.5–50.0 mg/L). In particular, our results showed that chitosan (5.0 mg/L) improved both biomass production and accumulation of silymarin from *S. marianum* cell suspension cultures. The resulting extracts also demonstrated their ability to act as antioxidant and anti-inflammatory supplements. Interestingly, by using a cell suspension system, the present research has the potential to scale up to the level of the bioreactor for enhanced production of silymarin-rich extracts and their possible commercial use.

## Figures and Tables

**Table 1:** Fresh weight (FW) and Dry weight (DW) of samples on different chitosan concentrations.

Elicitor Treatment	Fresh Weight (FW, g/L)	Dry Weight (DW, g/L)
MCH 1	64.8 ± 0.7 ^e^	6.1 ± 0.3 ^e^
MCH2	73.8 ± 1.2 ^d^	8.5 ± 0.1 ^d^
MCH3	82.3 ± 1.5 ^c^	10.9 ± 0.3 ^c^
MCH4	114.2 ± 0.4 ^b^	13.6 ± 1.3 ^b^
MCH5	123.3 ± 1.7 ^a^	17.7 ± 0.5 ^a^
MCH6	79.2 ± 0.3 ^c^	10.0 ± 1.2 ^c^
MCH7	47.7 ± 0.5 ^f^	5.5 ± 0.1 ^e^
MCH8	63.8 ± 0.1 ^e^	9.2 ± 1.7 ^cd^

Values are means ± SD of three independent replicates. MCH1: control; MCH2: 0.5 mg/L chitosan; MCH3: 1.0 mg/L chitosan; MCH4: 2.5 mg/L chitosan; MCH5: 5.0 mg/L chitosan; MCH6: 10.0 mg/L chitosan; MCH7: 25.0 mg/L chitosan; MCH8: 50.0 mg/L chitosan; Different letters represent significant differences between the various extraction conditions (*p* < 0.05).

**Table 2:** Silymarin quantification of suspension culture under different chitosan concentrations.

Compounds	Chitosan Treatments							
	MCH 1	MCH 2	MCH 3	MCH 4	MCH 5	MCH 6	MCH 7	MCH 8
Silybin A ^1^	0.6 ± 0.0 ^c^	1.2 ± 0.1 ^ab^	0.4 ± 0.0 ^e^	0.5 ± 0.1 ^de^	1.2 ± 0.1 ^a^	0.6 ± 0.0 ^cd^	0.6 ± 0.0 ^c^	1.1 ± 0.1 ^b^
Silybin B ^1^	3.5 ± 0.6 ^b^	6.3 ± 0.2 ^a^	2.5 ± 0.0 ^d^	2.8 ± 0.1 ^cd^	6.1 ± 0.2 ^a^	2.9 ± 0.1 ^bc^	3.2 ± 0.3 ^bc^	5.4 ± 1.0 ^a^
Isosilybin A ^1^	0.2 ± 0.0 ^ab^	0.2 ± 0.0 ^a^	0.2 ± 0.0 ^b^	0.2 ± 0.0 ^b^	0.2 ± 0.0 ^a^	0.2 ± 0.0 ^b^	0.2 ± 0.0 ^ab^	0.2 ± 0.0 ^a^
Isosilybin B ^1^	0.1 ± 0.00 ^b^	0.1 ± 0.0 ^a^	0.1 ± 0.0 ^e^	0.1 ± 0.0 ^de^	0.2 ± 0.0 ^a^	0.1 ± 0.0 ^d^	0.1 ± 0.0 ^c^	0.1 ± 0.0 ^ab^
Silychristin ^1^	0.5 ± 0.0 ^c^	0.7 ± 0.1 ^ab^	0.3 ± 0.0 ^f^	0.4 ± 0.0 ^e^	0.7 ± 0.0 ^a^	0.4 ± 0.0 ^de^	0.4 ± 0.0 ^d^	0.6 ± 0.1 ^b^
Isosilychristin ^1^	0.3 ± 0.0 ^b^	0.4 ± 0.0 ^a^	0.2 ± 0.0 ^b^	0.2 ± 0.0 ^b^	0.4 ± 0.0 ^ab^	0.2 ± 0.0 ^b^	0.3 ± 0.0 ^b^	0.4 ± 0.0 ^a^
Silydianin ^1^	0.7 ± 0.1 ^c^	1.0 ± 0.0 ^a^	0.6 ± 0.0 ^c^	0.7 ± 0.1 ^c^	1.0 ± 0.0 ^a^	0.7 ± 0.0 ^c^	0.7 ± 0.1 ^c^	0.9 ± 0.0 ^b^
Taxifolin ^1^	0.1 ± 0.0 ^bc^	0.1 ± 0.0 ^a^	0.0 ± 0.0 ^d^	0.0 ± 0.0 ^c^	0.1 ± 0.0 ^a^	0.0 ± 0.0 ^c^	0.0 ± 0.00 ^b^	0.1 ± 0.0 ^a^
Total Silymarin ^1^	5.9 ± 0.8 ^b^	9.9 ± 0.5 ^a^	4.3 ± 0.3 ^c^	4.8 ± 0.6 ^b^	9.8 ± 0.5 ^a^	5.0 ± 0.3 ^b^	5.4 ± 0.6 ^b^	8.7 ± 1.3 ^a^

^1^ Expressed in mg/g DW; MCH1: control; MCH2: 0.5 mg/L chitosan; MCH3: 1.0 mg/L chitosan; MCH4: 2.5 mg/L chitosan; MCH5: 5.0 mg/L chitosan; MCH6: 10.0 mg/L chitosan; MCH7: 25.0 mg/L chitosan; MCH8: 50.0 mg/L chitosan; Values are means ± SD of three independent replicates. Different letters represent significant differences between the various extraction conditions (*p* < 0.05).

**Table 3:** Different antioxidant activities of SMCE.

Treatment	Antioxidant Assays			
	DPPH ^1^	FRAP ^2^	ABTS ^2^	ROS ^3^
**MCH1**	87.3 ± 0.9 ^b^	326.8 ± 3.7 ^bc^	703.5 ± 4.2 ^d^	71.4 ± 1.2 ^c^
**MCH2**	81.2 ± 2.0 ^cd^	334.5 ± 3.3 ^a^	741.5 ± 4.4 ^a^	74.5 ± 1.9 ^bc^
**MCH3**	87.2 ± 1.2 ^bc^	330.9 ± 3.3 ^ab^	720.1 ± 4.3 ^c^	76.8 ± 1.2 ^ab^
**MCH4**	78.2 ± 1.1 ^d^	332.9 ± 3.2 ^ab^	730.5 ± 4.1 ^b^	76.4 ± 1.5 ^ab^
**MCH5**	90.4 ± 1.0 ^a^	330.2 ± 3.3 ^ab^	730.4 ± 4.6 ^b^	78.8 ± 1.3 ^a^
**MCH6**	84.1 ± 1.3 ^c^	332.4 ± 3.2 ^ab^	729.5 ± 4.2 ^bc^	78.4 ± 1.2 ^a^
**MCH7**	83.3 ± 1.1 ^c^	320.9 ± 3.2 ^c^	719.3 ± 5.0 ^c^	76.3 ± 1.1 ^ab^
**MCH8**	89.5 ± 0.9 ^ab^	331.7 ± 3.4 ^ab^	733.9 ± 4.3 ^ab^	71.6 ± 1.2 ^c^

^1^ Expressed in % of free radical scavenging activity (%FRSA); ^2^ Expressed in µM of Trolox C equivalent antioxidant activity (µM TEAC); ^3^ Expressed in % inhibition of the cellular production of reactive oxygen and nitrogen species (RO/NS). MCH1: control; MCH2: 0.5 mg/L chitosan; MCH3: 1.0 mg/L chitosan; MCH4: 2.5 mg/L chitosan; MCH5: 5.0 mg/L chitosan; MCH6: 10.0 mg/L chitosan; MCH7: 25.0 mg/L chitosan; MCH8: 50.0 mg/L chitosan; Values are means ± SD of three independent replicates. Different letters represent significant differences between the various extraction conditions (*p* < 0.05).

**Table 4:** Different anti-inflammatory activities of SMCE.

Treatment		% Inhibition		
	COX1 ^1^	COX2 ^1^	15-LOX ^1^	sPLA2 ^1^
MCH1	14.6 ± 1.2 ^b^	20.5 ± 1.0 ^c^	20.5 ± 1.2 ^c^	21.4 ± 1.3 ^cd^
MCH2	22.3 ± 1.0 ^a^	31.2 ± 1.0 ^a^	35.4 ± 1.3 ^a^	34.2 ± 0.9 ^a^
MCH3	13.4 ± 1.3 ^b^	17.2 ± 1.2 ^d^	20.1 ± 1.3 ^cd^	22.4 ± 1.2 ^c^
MCH4	12.9 ± 1.2 ^b^	18.2 ± 1.1 ^cd^	20.5 ± 1.1 ^c^	22.4 ± 1.5 ^c^
MCH5	20.2 ± 1.3 ^a^	29.9 ± 1.3 ^a^	30.1 ± 1.6 ^b^	28.8 ± 1.3 ^b^
MCH6	12.4 ± 1.2 ^b^	22.1 ± 1.6 ^bc^	17.5 ± 1.2 ^d^	18.4 ± 1.2 ^dc^
MCH7	20.9 ± 1.2 ^a^	23.3 ± 1.1 ^b^	19.3 ± 1.0 ^cd^	16.3 ± 1.1 ^e^
MCH8	21.7 ± 1.4 ^a^	30.5 ± 1.3 ^a^	33.9 ± 1.3 ^a^	31.6 ± 1.2 ^ab^

^1^ Expressed in % of inhibition relative to control conditions (addition of the same volume of extraction solvent); MCH1: control; MCH2: 0.5 mg/L chitosan; MCH3: 1.0 mg/L chitosan; MCH4: 2.5 mg/L chitosan; MCH5: 5.0 mg/L chitosan; MCH6: 10.0 mg/L chitosan; MCH7: 25.0 mg/L chitosan; MCH8: 50.0 mg/L chitosan; Values are means ± SD of three independent replicates. Different letters represent significant differences between the various extraction conditions (*p* < 0.05).

**Table 5:** Different tags and concentrations of chitosan used.

Elicitor	Tags	Concentration (s)
No elicitor	MCH1	Control
Chitosan	MCH2	0.5 mg/L
	MCH3	1.0 mg/L
	MCH4	2.5 mg/L
	MCH5	5.0 mg/L
	MCH6	10.0 mg/L
	MCH7	25.0 mg/L
	MCH8	50.0 mg/L

## Data Availability

All the data are included in the present study and the online associated supplementary materials.

## Supplementary Materials

The following are available online, Figure S1: Aspects of cell suspension cultures of *S. marianum* submitted to different concentrations of chitosan. Figure S2: Loading scores of the first (PC1) and second (PC2) axis of the principal component analysis of the parameters measured in extract of cell suspension cultures of *S. marianum* in response to chitosan elicitation; Table S1: Actual values for PCC (Pearson correlation coefficient) presented in Figure 2 showing the relation between the main phytochemicals and the biological activities (antioxidant and anti-inflammatory) of extracts of cell suspension cultures of *S. marianum* in response to chitosan elicitation.

## Sample Availability

Samples are available from the authors upon request.
