# Supplementary material for: Chitosan Elicitation Impacts Flavonolignan Biosynthesis in Silybum marianum (L.) Gaertn Cell Suspension and Enhances Antioxidant and Anti-Inflammatory Activities of Cell Extracts
Source: Molecules. 2021 Feb 3;26(4):791. doi: 10.3390/molecules26040791 (PMC7913645; doi:10.3390/molecules26040791)
Supplement: Supplementary file 1 [file molecules-26-00791-s001.pdf]

molecules MDPI

SUPPLEMENTARY MATERIALS

Chitosan Elicitation Impacts Flavonolignan Biosynthesis in *Silybum marianum* (L.) Gaertn Cell Suspension and Enhances Antioxidant and Anti-inflammatory Activities of Cell Extracts

Muzamil Shah[1], Hasnain Jan[1], Samantha Drouet[2], Duangjai Tungmunnithum[3], Jafir Hussain Shirazi[4], Christophe Hano[2*], Bilal Haider Abbasi[1*]

[1] Department of Biotechnology, Quaid-i-Azam University, Islamabad-45320, Pakistan; mshah@bs.qau.edu.pk (M.S.); rhasnain849@gmail.com (H.J.); bhabbasi@qau.edu.pk (B.H.A.)
[2] University of Orleans, Laboratoire de Biologie des Ligneux et des Grandes Cultures (LBLGC), INRAE USC1328, F28000 Chartres, France; samantha.drouet@univ-orleans.fr (S.D.); hano@univ-orleans.fr (C.H.)
[3] Department of Pharmaceutical Botany, Faculty of Pharmacy, Mahidol University, 447 Sri-Ayuthaya Road, Rajathevi, Bangkok 10400, Thailand; duangjai.tun@mahidol.ac.th (D.T.)
[4] Islamia University of Bahawalpur, Bahawalpur, Pakistan; jafir.shirazi@iub.edu.pk (J.H.S.)
* Correspondence: bhabbasi@qau.edu.pk (B.H.A.); hano@univ-orleans.fr (C.H.) Tel: +33-77-698-41-48 (B.H.A.); +33-237-309-753 (C.H.)

Supplementary Materials List:

Figure S1. Aspects of cell suspension cultures of *S. marianum* submitted to different concentrations of chitosan.

Figure S2: Loading scores of the first (PC1) and second (PC2) axis of the principal component analysis of the parameters measured in extract of cell suspension cultures of *S. marianum* in response to chitosan elicitation.

Table S1: Actual values for PCC (Pearson correlation coefficient) presented in Figure 4 showing the relation between the main phytochemicals and the biological activities (antioxidant and anti-inflammatory) of extracts of cell suspension cultures of *S. marianum* in response to chitosan elicitation.

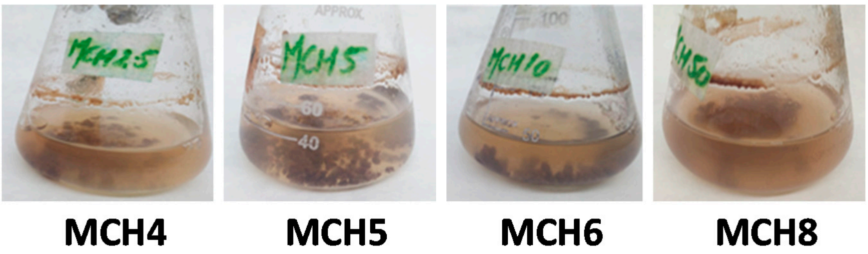

Figure S1. Aspects of cell suspension cultures of *S. marianum* submitted to different concentrations of chitosan (MCH4: 2.5 mg/L chitosan; MCH5: 5.0 mg/L chitosan; MCH6: 10.0 mg/L chitosan; MCH8: 50.0 mg/L chitosan).

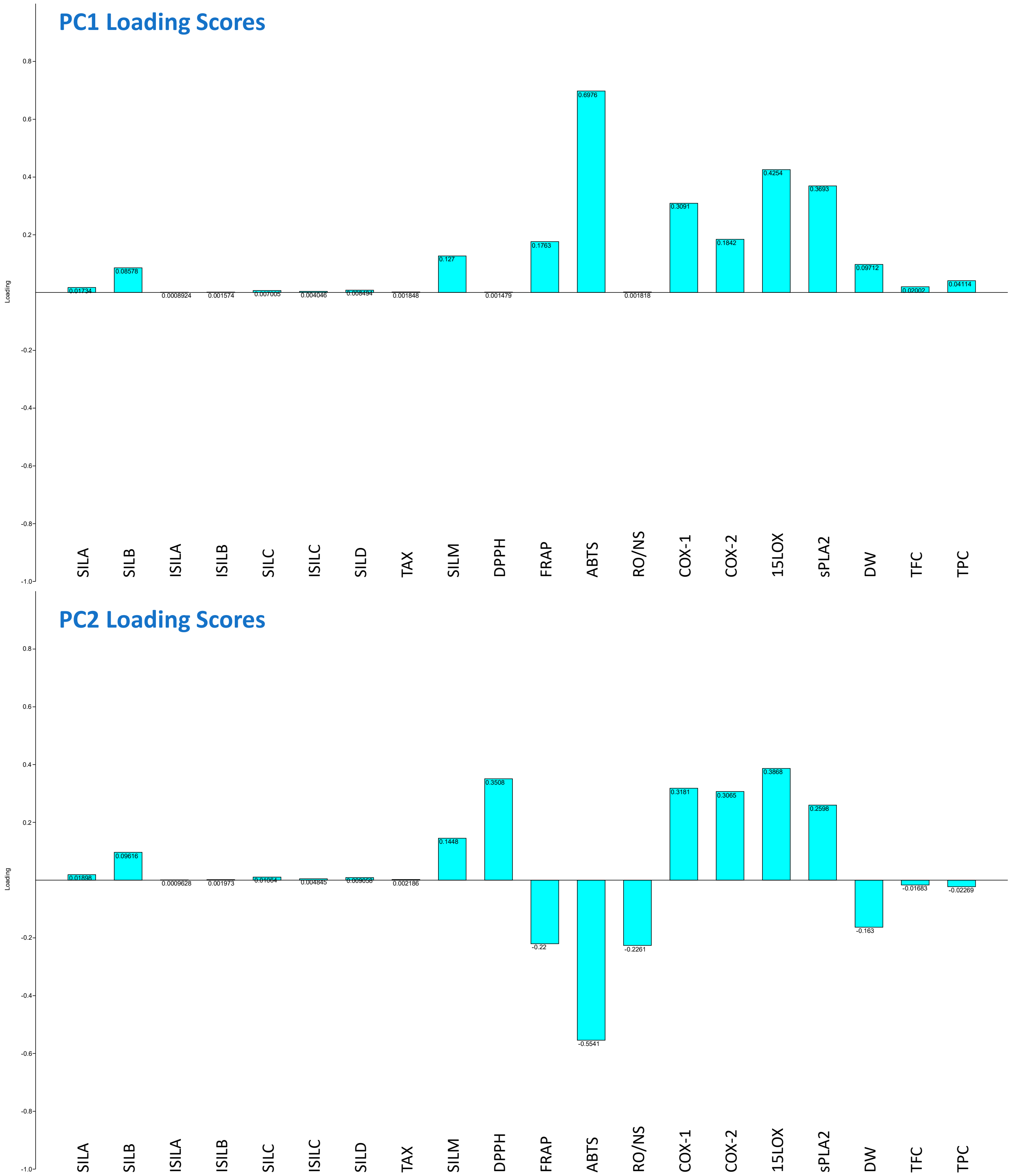

Figure S2: Loading scores of the first (PC1) and second (PC2) axis of the principal component analysis of the parameters measured in extract of cell suspension cultures of *S. marianum* in response to chitosan elicitation.

Phytochemicals: SILA: silybin A; SILB: silybin B; ISILA: isosilybin A; ISILB: isosilybin B; SILC: silychristin; ISILC: isosilychristin; SILD: silydianin; TAX: taxifolin; SILM: silymarin; TFC: total flavonoid content; TPC: total phenolic content. **Antioxidants assays:** DPPH: 2,2-diphenyl-1-picrylhydrazyl *in vitro* antioxidant assay; FRAP: ferric reducing antioxidant power *in vitro* antioxidant assay. ABTS: 2,2-azinobis-3-ethylbenzthiazoline-6-sulphonic acid *in vitro* antioxidant assay; RO/NS: cellular antioxidant assay (reactive of oxygen and nitrogen species). Anti-inflammatory: COX-1: cyclooxygenase 1 inhibition; COX-2: cyclooxygenase 2 inhibition; 15LOX: 15-lipoxygenase inhibition; sPLA2: secretory phospholipase A2 inhibition. **Biomass:** DW: dry weight.

Table S1: Actual values for PCC (Pearson correlation coefficient) presented in Figure 4 showing the relation between the main phytochemicals and the biological activities (antioxidant and anti-inflammatory) of extracts of cell suspension cultures of *S. marianum* in response to chitosan elicitation.

	SILA	SILB	ISILA	ISILB	SILC	ISILC	SILD	TAX	SILM	**TPC**	**TFC**
**DPPH**	*0.365* *ns*	*0.330* *ns*	*0.357* *ns*	*0.289* *ns*	*0.356* *ns*	*0.307* *ns*	*0.302* *ns*	*0.415* *ns*	*0.335* *ns*	*-0.101* *ns*	*-0.022* *ns*
**FRAP**	*0.287* *ns*	*0.301* *ns*	*0.266* *ns*	*0.236* *ns*	*0.213* *ns*	*0.313* *ns*	*0.267* *ns*	*0.253* *ns*	*0.291* *ns*	*0.344* *ns*	*0.756* *ns*
**ABTS**	*0.565* *ns*	*0.558* *ns*	*0.565* *ns*	*0.482* *ns*	*0.418* *ns*	*0.535* *ns*	*0.571* *ns*	*0.555* *ns*	*0.551* *ns*	0.782 *	0.815 *
**ROS/RNS**	*-0.152* *ns*	*-0.183* *ns*	*-0.097* *ns*	*-0.139* *ns*	*-0.237* *ns*	*-0.254* *ns*	*-0.140* *ns*	*-0.228* *ns*	*-0.181* *ns*	*0.452* *ns*	*0.268* *ns*
**COX1**	0.960 *	0.950 *	0.970 *	0.913 *	0.918 *	0.945 *	0.955 *	0.959 *	0.952 *	*0.392* *ns*	*0.464* *ns*
**COX2**	0.802 ***	0.810 ***	0.805 ***	0.781 ***	0.770 ***	0.801 ***	0.825 ***	0.828 ***	0.809 ***	*0.437* *ns*	*0.298* *ns*
**15-LOX**	0.927 ***	0.942 ***	0.897 **	0.882 **	0.878 **	0.946 ***	0.926 ***	0.937 ***	0.936 ***	*0.490* *ns*	*0.660* *ns*
**SPLA2**	0.853 **	0.877 **	0.814 *	0.815 *	0.808 *	0.886 **	0.850 **	0.851 **	0.868 **	*0.473* *ns*	*0.751* *ns*

Significance level: * $p < 0.05$, ** $p < 0.01$, *** $p < 0.001$.
Phytochemicals: SILA: silybin A; SILB: silybin B; ISILA: isosilybin A; ISILB: isosilybin B; SILC: silychristin; ISILC: isosilychristin; SILD: silydianin; TAX: taxifolin; SILM: silymarin; TFC: total flavonoid content; TPC: total phenolic content. **Antioxidants assays:** DPPH: 2,2-diphenyl-1-picrylhydrazyl *in vitro* antioxidant assay; FRAP: ferric reducing antioxidant power *in vitro* antioxidant assay. ABTS: 2,2-azinobis-3-ethylbenzthiazoline-6-sulphonic acid *in vitro* antioxidant assay; RO/NS: cellular antioxidant assay (reactive of oxygen and nitrogen species). Anti-inflammatory: COX-1: cyclooxygenase 1 inhibition; COX-2: cyclooxygenase 2 inhibition; 15LOX: 15-lipoxygenase inhibition; sPLA2: secretory phospholipase A2 inhibition.
Biomass: DW: dry weight.
